# The Barrier Inhomogeneity and the Electrical Characteristics of W/Au *β*-Ga_2_O_3_ Schottky Barrier Diodes

**DOI:** 10.3390/mi16040369

**Published:** 2025-03-25

**Authors:** Lei Xie, Tao Zhang, Shengrui Xu, Huake Su, Hongchang Tao, Yuan Gao, Xu Liu, Jincheng Zhang, Yue Hao

**Affiliations:** 1 State Key Laboratory of Wide-Bandgap Semiconductor Devices and Integrated Technology, School of Microelectronics, Xidian University, Xi’an 710071, China; 22111213660@stu.xidian.edu.cn (L.X.); huakexidian@126.com (H.S.); taohongchang@xidian.edu.cn (H.T.); 15619217227@163.com (Y.G.); xliu23@stu.xidian.edu.cn (X.L.); jchzhang@xidian.edu.cn (J.Z.); yhao@xidian.edu.cn (Y.H.); 2 Shaanxi Engineering Technology and Research Center of High-Power Semiconductor Lighting, Xi’an 710071, China

**Keywords:** Ga_2_O_3_ SBDs, W/Au Schottky contact, barrier inhomogeneity

## Abstract

In this work, the electrical properties of the Ga_2_O_3_ Schottky barrier diodes (SBDs) using W/Au as the Schottky metal were investigated. Due to the 450 °C post-anode annealing (PAA), the reduced oxygen vacancy defects on the *β*-Ga_2_O_3_ surface resulted in the improvement in the forward characteristics of the W/Au Ga_2_O_3_ Schottky diode, and the breakdown voltage was significantly enhanced, increasing by 56.25% from 400 V to 625 V after PAA treatment. Additionally, the temperature dependence of barrier heights and ideality factors was analyzed using the thermionic emission (TE) model combined with a Gaussian distribution of barrier heights. Post-annealing reduced the apparent barrier height standard deviation from 112 meV to 92 meV, indicating a decrease in barrier height fluctuations. And the modified Richardson constants calculated for the as-deposited and annealed samples were in close agreement with the theoretical value, demonstrating that the barrier inhomogeneity of the W/Au Ga_2_O_3_ SBDs can be accurately explained using the TE model with a Gaussian distribution of barrier heights.

## 1. Introduction

With the development of technology, there is a growing demand for higher operating voltages, ambient temperatures, and more efficient energy conversion. Materials such as BN, AlN, Ga_2_O_3_, and diamond, known for their wide bandgap, ultra-high breakdown voltage, and exceptional chemical and mechanical stability, have increasingly gained attention [1,2,3,4]. Among them, *β*-Ga_2_O_3_ is the only material that can be grown through the melt growth method [5], and high-quality *β*-Ga_2_O_3_ native bulk crystal substrates are available through single crystal growth. Currently, 4-inch *β*-Ga_2_O_3_ single crystal substrates have been commercially mass-produced [6]. In addition, *β*-Ga_2_O_3_ also boasts an extremely high critical breakdown field [7], a much smaller power loss [8] compared to silicon and gallium nitride [9], and higher electron saturation velocity [10], making it promising for use in the next generation of commercial power conversion and radio frequency electronic devices [11,12].

Various structures of *β*-Ga_2_O_3_ SBDs have been extensively investigated [13]. One of the primary design considerations for Schottky diodes is achieving homogeneous Schottky contacts to predict the diode’s electrical performance under varying temperatures and voltages [14]. However, Schottky contact inhomogeneity is commonly observed in *β*-Ga_2_O_3_. Similar to that in other semiconductors such as GaN and SiC [15,16,17], the Schottky contact inhomogeneity can lead to the strong temperature dependence of the Schottky barrier height (Ф_b_) and the ideality factor, and the experimental Richardson constant is significantly lower than the theoretical value (A** = 41.1 A/cm^2^K^2^) [18]. The barrier height and ideality factor vary widely with temperature being sometimes attributed to large-density of surfaces states, resulting in Fermi-level pinning effect at metal/*β*-Ga_2_O_3_ interface [19]. The origin of large-density surface states is likely to be associated with surface contaminants and surface defects like grain boundaries, multiple phases, facets, vacancies, and different threading dislocations [20]. The large density states on the surface serve as the patch of low barrier height regions, resulting in bad device characteristics and barrier inhomogeneity in Schottky diodes [21]. In the past several decades, the barrier inhomogeneity has been extensively investigated, and many types of barrier height distribution functions have been proposed to describe the barrier inhomogeneity. So far, the barrier inhomogeneity of metals with high work function (such as Ni, Pt, Pd) on *β*-Ga_2_O_3_ SBDs has been explained on basis of thermionic emission theory [22,23,24]. However, the barrier inhomogeneity of metals with lower work function on *β*-Ga_2_O_3_ SBDs, like tungsten, has not yet been explored.

In this work, we report a detailed analysis on electrical characteristics and barrier inhomogeneity of vertical *β*-Ga_2_O_3_ SBDs using W/Au as the Schottky metal. The capacitance–voltage (*C*-*V*) characteristics, the forward current density–voltage (*J*-*V*) characteristics, and the reverse breakdown voltage (*BV*) were investigated. An improvement in the diode characteristics is observed in the W/Au Ga_2_O_3_ SBDs following PAA treatment. Furthermore, the barrier inhomogeneity was observed from the current–voltage (*I*-*V*) characteristics of the Schottky diodes at varied temperatures ranging from 300 K to 450 K. Based on the thermionic emission model combined with Gaussian distribution of barrier heights, the temperature dependence of barrier heights and ideality factors was explained, and the barrier height fluctuation was reduced after anode metal annealing. Meanwhile, the modified Richardson constants are close to the theoretical value, demonstrating that the barrier height distribution of the W/AuGa_2_O_3_ Schottky contacts is followed by Gaussian distribution.

## 2. Device Structure and Process

**Figure 1:**
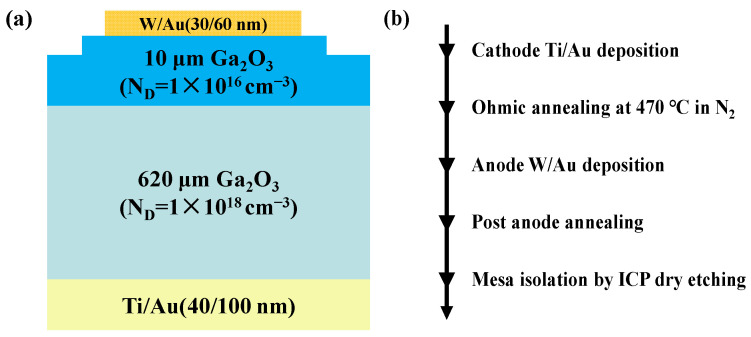
(**a**) Schematic cross section structure of the fabricated W/Au Ga_2_O_3_ SBDs; (**b**) the fabrication process of the Ga_2_O_3_ SBDs.

The schematic cross section structure of the fabricated Ga_2_O_3_ SBDs and the fabrication process are shown in Figure 1a and b, respectively. The substrate material was Sn-doped (*n* = 1 × 10^18^ cm^−3^) *β*-Ga_2_O_3_ single crystal substrates grown by the edge-defined film-fed growth method, then a 10-μm Si-doped (*n* = 1 × 10^16^ cm^−3^) epitaxial layer is grown by halide vapor phase epitaxy on top of *β*-Ga_2_O_3_ substrates. Prior to device fabrication, the wafer was cleaned with piranha solution and then soaked in dilute buffered oxide etch for 10 min to remove the surface residual contaminants. The full-area back cathode metal Ti/Au (40/100 nm) was deposited by electron-beam evaporation, followed by rapid annealing process at 470 °C in N_2_ for 60 s to form ohmic contacts. Then, the Schottky contacts were formed by the deposition of W/Au (30/60 nm), and 450 °C post-anode annealing for 2 min was performed in N_2_. Lastly, the mesa isolation was formed by inductively coupled plasma (ICP) dry etching with 1 μm depth. The *C-V* characteristics, the *I-V* measurement at varied temperatures ranging from 300 K to 450 K, and the reverse *BV* characteristics of the W/Au-Ga_2_O_3_ SBDs were taken with the Keysight B1505A semiconductor parameter analyzer, and the four-point probe testing method was used to reduce probe resistance.

## 3. Results and Discussion

**Figure 2:**
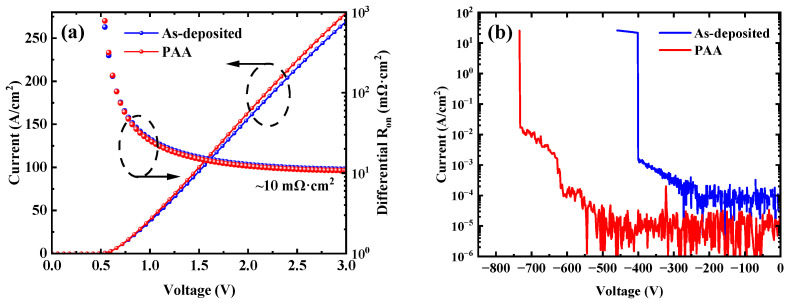
(**a**) Forward *J*-*V* characteristics and (**b**) reverse *BV* characteristics of the W/Au Ga_2_O_3_ SBDs for the as-deposited sample and the sample with PAA.

Figure 2a shows the forward *J*-*V* characteristics of the W/Au Ga_2_O_3_ SBDs for the as-deposited sample and the sample with PAA. The barrier heights and ideality factors were extracted by fitting the linear regime of *I*-*V* curves using the thermionic emission model. After post-anode annealing, the barrier height increases from 0.85 eV to 0.88 eV and the ideality factor decreases from 1.21 to 1.14, respectively. In addition, as shown in Figure 2b, the breakdown voltage at the current density of 1 mA/cm^2^ increases by 56.25%, rising from 400 V to 625 V after the PAA treatment. On the surface of the Ga_2_O_3_ material, surface defects such as oxygen vacancies can lead to a large number of surface states, which can seriously affect the device characteristics [25]. The improvement of W/Au Ga_2_O_3_ diode characteristics after PAA treatment can be attributed to the reduction of surface defects, with further analysis shown below.

**Figure 3:**
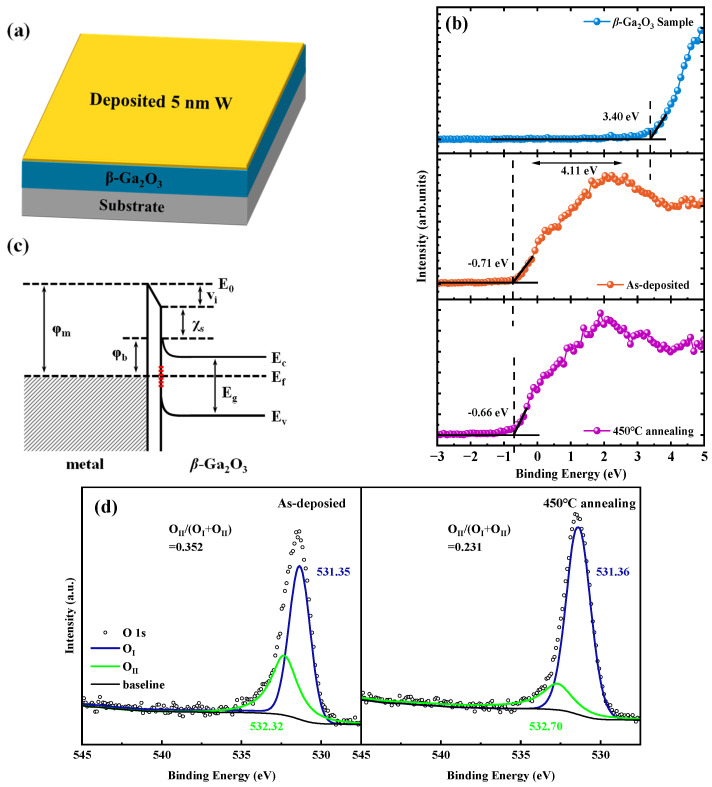
(**a**) 5 nm thick W metal was deposited on the *β*-Ga_2_O_3_ sample surface; (**b**) XPS valence band plots for the *β*-Ga_2_O_3_ sample, as-deposited sample with 5 nm W metal film on Ga_2_O_3_, and the sample-deposited 5 nm W and annealed for 2 min at 450 °C in N_2_ atmosphere; (**c**) the barrier diagram of the metal in contact with *β*-Ga_2_O_3_; (**d**) the O 1s core level spectra for the sample-deposited 5 nm W and the sample-deposited 5 nm W with 450 °C annealing.

To investigate the improvement of W/Au Ga_2_O_3_ diode characteristics following PAA treatment, X-ray photoelectron spectroscopy (XPS) measurements were performed on the *β*-Ga_2_O_3_ samples. As shown in Figure 3a, a 5 nm thick W metal layer was deposited on the surface of the *β*-Ga_2_O_3_ sample. Prior to the XPS measurement, the W-deposited sample was etched with Ar^+^ plasma to enhance the accuracy of the results. Figure 3b presents the XPS valence band plots for the *β*-Ga_2_O_3_ sample, the as-deposited sample with the 5 nm W metal film, and the sample-deposited 5 nm W and with 450 °C annealing. According to Figure 3c, the barrier height (*ϕ_b_*) of the metal contact with *β*-Ga_2_O_3_ can be expressed as *ϕ_b_ = ϕ_m_ – χ_s_ – eV_i_*, where the surface potential (*V_i_*) is induced by the surface states located within the bandgap [26]. As illustrated in Figure 3b, the valence band maximum of the sample treated at 450 °C increases from −0.71 eV to −0.66 eV compared to the as-deposited sample. This difference indicates that the surface potential is reduced by about 0.05 eV after the 450 °C annealing, which is consistent with the increased barrier height extracted from the *I-V* characteristics. In addition, Figure 3d shows the O 1s core level spectra, which can be fitted into two peaks. The peaks around 531 eV (O_I_) and 533 eV (O_II_) are attributed to O^2−^ ions and oxygen vacancies, respectively [27], and the proportion of O_II_ represents the relative concentration of oxygen vacancies on the *β*-Ga_2_O_3_ surface. As shown in Figure 3d, the proportion of O_II_ decreases from 35.2% to 23.1% after the 450 °C annealing treatment, indicating that the oxygen vacancies on the *β*-Ga_2_O_3_ surface are reduced. These oxygen vacancy defects act as surface states, whose reduction can lead to a decrease in the surface potential and an increase in the barrier height. At the same time, the oxygen vacancy defects and gallium–oxygen vacancy pairs are the origin of the donor-type defects and acceptor-type defects in the Ga_2_O_3_ material bandgap, respectively [28], where the reduction of oxygen vacancy defects on the *β*-Ga_2_O_3_ surface can also reduce the paths of electron assisted tunnelling through these defects [29], resulting in a decreased leakage current under reverse bias and an increased breakdown voltage.

**Figure 4:**
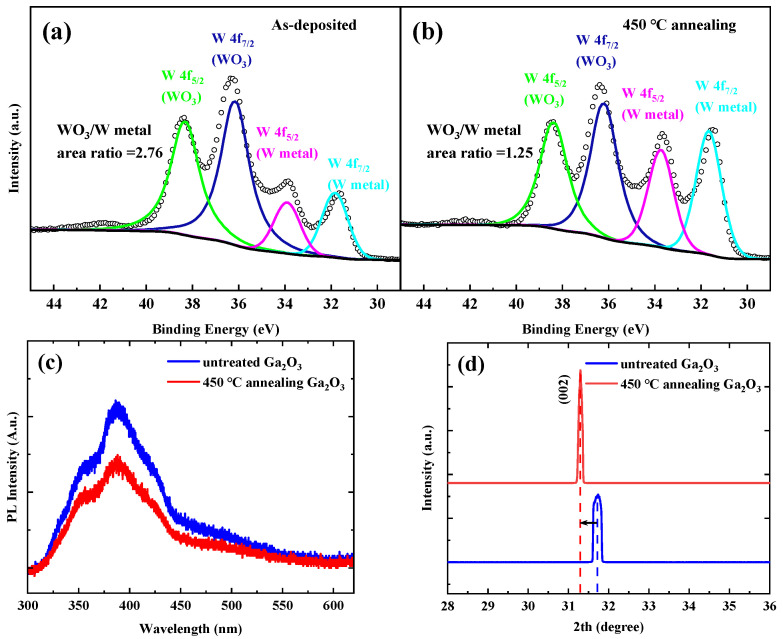
The W 4f core level spectra for (**a**) the sample-deposited 5 nm W and (**b**) the sample-deposited 5 nm W with 450 °C annealing; (**c**) the PL spectra and (**d**) the 2th scan spectra for the untreated Ga_2_O_3_ epitaxial material and the Ga_2_O_3_ epitaxial material with 450 °C annealing.

The reduction of oxygen vacancies on the *β*-Ga_2_O_3_ surface can be attributed to two aspects. On the one hand, the W metal has a low chemical affinity (i.e., lower oxide stability and no intermetallic compounds) with Ga_2_O_3_, so that WO_x_ oxides are easily reduced to the W native metal during annealing [30]. Figure 4a and b show the W 4f spectra for the sample-deposited 5 nm W and the sample-deposited 5 nm W with 450 °C annealing. As shown in Figure 4a and Figure 4b, the peaks around 36.1 eV and 31.6 eV are attributed to WO_3_ and W metal, respectively. The W 4f core level spectra were deconvoluted using a doublet of two Gaussian peaks of equal width corresponding to the W 4f_7/2_ and W 4f_5/2_ spin-orbit components [31]. After annealing, the ratio of WO_3_ to W metal decreases from 2.76 to 1.25, indicating that WO_3_ oxides are partially reduced to the W native metal during annealing. Meanwhile, the excess oxygen atoms diffuse into the Ga_2_O_3_ material surface in the presence of pure nitrogen, thus reducing the surface oxygen vacancies. On the other hand, during the annealing process in nitrogen atmosphere, the oxygen atoms previously present in the Ga_2_O_3_ material interstitial positions have the probability to migrate to the correct position, which can also result in the reduction of oxygen vacancy and the improvement of Ga_2_O_3_ material crystal quality [32]. Figure 4c shows the PL spectra for the untreated Ga_2_O_3_ epitaxial material and the Ga_2_O_3_ epitaxial material with 450 °C annealing. As shown in Figure 4c, a significant emission peak at about 387 nm and a broad blue-green emission band are observed. In various Ga_2_O_3_ materials, the broad 350–620 nm emission band is common, and the origin can be ascribed to the emission recombination of electron and hole pairs between donor-type oxygen vacancy defects and acceptor-type gallium–oxygen vacancy pairs [28]. The PL emission intensity of the Ga_2_O_3_ epitaxial material decreases after annealing, demonstrating that the oxygen vacancies in the Ga_2_O_3_ material are reduced after annealing. Additionally, Figure 4d shows the XRD images for the untreated Ga_2_O_3_ epitaxial material and the Ga_2_O_3_ epitaxial material with 450 °C annealing in nitrogen atmosphere. The XRD results show that the intensity of the (002) diffraction peak (about 31.5°) is enhanced in the Ga_2_O_3_ epitaxial material with annealing. This indicates that the crystal quality of Ga_2_O_3_ epitaxial material is improved after annealing at 450 °C in N_2_ atmosphere, and the stress release leads to the location of the (002) peak shift [33]. These results are consistent with the previous conjectures.

**Figure 5:**
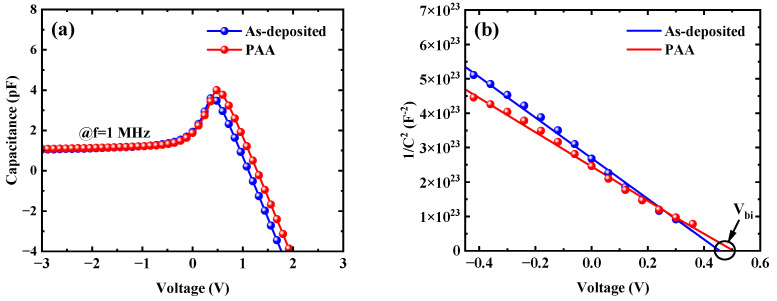
(**a**) *C-V* characteristics and (**b**) 1/C^2^ versus voltage V plots for the as-deposited sample and the sample with 450 °C PAA.

Figure 5a shows the *C-V* characteristics of the W/Au SBDs as a frequency of 1 MHz for the as-deposited sample and the sample with PAA, and it conforms to the typical behavior of vertical Schottky barrier diodes. As the anode voltage increases, majority carrier electrons accumulate gradually, leading to an increase of the capacitance [34]. Meanwhile, the capacitance 1/*C*^2^ versus applied voltage plots is shown in Figure 5b, and the relationship of the 1/*C*^2^*-V* is given by the following Equation [35]:

(1) $$\frac{1}{C^{2}}=\frac{2}{A^{2}q\varepsilon_{s}N_{D}}(\Phi_{b}-V_{n}-V\frac{k_{B}T}{q})$$

where *C* is the capacitance, *A* is the Schottky electrode area, *ε_s_* is the dielectric constant of *β*-Ga_2_O_3_, *N_D_* is the effective carrier concentration, *Ф_b_* is the effective barrier height, and *V_n_* is the voltage corresponding to the difference between the conduction band edge (*E_c_*) and the Fermi level (*E_f_*) in the flat-band condition, which can be expressed as:

(2) $$V_{n}=E_{c}-E_{f}=\frac{k_{B}T}{q}\ln\frac{N_{c}}{N_{D}}$$

where *N_c_* is the effective state density of the conduction band edge, which is given by:

(3) $$N_{c}=2{\left(\frac{2\pi m^{*}k_{B}T}{h^{2}}\right)}^{3/2}$$

where m* is the effective mass of electron, and *h* is the Planck constant. By combining Equations (1) and (2), the effective carrier concentration and barrier height can be calculated from the 1/*C*^2^-*V* plots as follows:

(4) $$N_{D}=\frac{2}{A^{2}q\varepsilon_{s}}\frac{1}{K_{c}}$$

(5) $$\Phi_{b,0}=V_{\mathrm{bi}}+V_{n}+\frac{k_{B}T}{q}$$

where *K_c_* and *V_bi_* are the slope of the 1/*C*^2^*-V* plots and the x-axis intercept of the 1/*C*^2^*-V* plots, respectively. As shown in Figure 5b, the calculated effective carrier concentrations are approximately 1.2 × 10^15^ cm^−3^, and the *Ф_b_*_,0_ values obtained from C-V measurements are 0.72 eV for the as-deposited sample and 0.76 eV for the sample with PAA, where the calculated barrier heights are consistent with the results reported in the literature [36].

**Figure 6:**
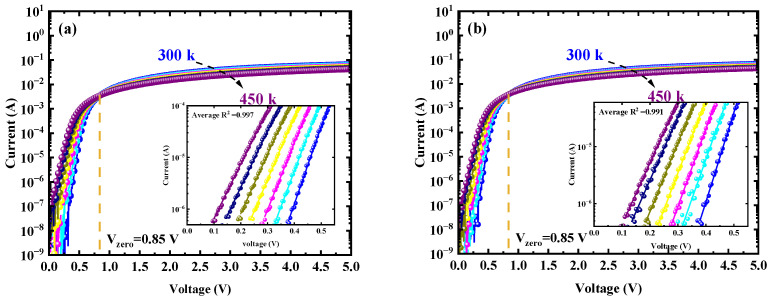
The *I-V* characteristics of the W/Au Ga_2_O_3_ Schottky diodes at varied temperatures ranging from 300 K (blue) to 450 K (purple). (**a**) The as-deposited sample; (**b**) the sample with 450 °C PAA, and the inset is the linear region of the curves where a linear fit to the data is applied.

In order to investigate the barrier inhomogeneity of the W/Au Ga_2_O_3_ SBDs, the *I-V* characteristics of the Schottky diodes at varied temperatures ranging from 300 K to 450 K, as shown in Figure 6, were analyzed. A zero-crossing voltage V_zero_ was observed at 0.85 V in the as-deposited sample and the sample with PAA, which may be due to two different conduction mechanisms in the temperature dependence of the *I-V* characteristics [37]. When the forward voltage is below 0.85 V, electron thermionic emission is dominated between the W/Au metal and Ga_2_O_3_ contacts, with more electrons acquiring sufficient energy to overcome the Schottky barrier as the temperature rises, leading to an increase in current. Conversely, when the voltage exceeds 0.85 V, electron transport occurs within the drift layer, whereas the current is governed by the mobility of electrons in the drift layer. In this region, as the temperature rises, electron mobility decreases, resulting in a reduction in current.

Based on the TE theory, the *I-V* characteristics are expressed by the following relationships [22,38]:

(6) $$I=AA^{**}T^{2}\exp(-\frac{q\Phi_{b}}{\mathrm{kT}})[\exp(\frac{\mathrm{qV}}{\mathrm{kT}}-1)]$$

and

(7) $$I_{s}=AA^{**}T^{2}\mathrm{ex}p\left(-\frac{q\Phi_{b,0}}{\mathrm{kT}}\right)$$

where *A* is the area of the Schottky contact, A** is the Richardson constant, with a theoretical value of 41.1 A/cm^2^K^2^ for *β*-Ga_2_O_3_, (calculated using m* = 0.34m_0_ from first-principles calculations), while *k*, *q*, *T*, and *I_s_* are the Boltzmann constant, electron charge, absolute temperature, and saturation current density, respectively. The Schottky barrier height *qФ_b_* is the V-dependent Schottky barrier height and can be expressed as Ф_b_ = Ф_b,0_ + *β*V, where *β* is the positive constant. Substituting this into Equation (6), the current can be rewritten as:

(8) $$I=AA^{**}T^{2}\mathrm{ex}p\left(-\frac{q\Phi_{b,0}+\beta V}{\mathrm{kT}}\right)[\exp(\frac{\mathrm{qV}}{\mathrm{kT}}-1)]\;=I_{s}\mathrm{ex}p\left(\frac{\mathrm{qV}}{\eta \mathrm{kT}}\right)[1-\exp(-\frac{\mathrm{qV}}{\mathrm{kT}})]$$

where *η* = 1/1 – *β*, and *η* is the ideality factor. When V > 3kq/T, Equation (8) simplifies to:

(9) $$I=I_{s}\exp\left(\frac{\mathrm{qV}}{\eta \mathrm{kT}}\right)$$

**Figure 7:**
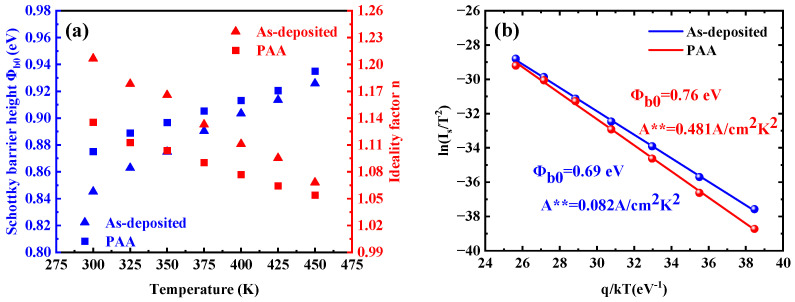
(**a**) Extracted barrier height and ideality factor in W/Au Ga_2_O_3_ SBDs at temperatures 300–450 K and (**b**) the conventional Richardson’s plot ln(I_s_/T^2^) versus q/kT for the as-deposited sample and the sample with 450 °C PAA.

According to Equation (9), the saturation current density *I_s_* and the ideality factor *η* can be extracted from the slope and *y*-intercept *y*-intercept and slope of the *ln*(*I*)-*V* plots, respectively. In addition, the barrier height *Ф_b_* was calculated from Equation (7). As shown in the inset of Figure 6, the barrier height was extracted by selecting a segment of the semi-logarithmic plot (approximately 0.1–0.5 V) within the linear region for linear fitting, from which the slope of the fitted Equation was determined. The obtained average goodness-of-fit metric R^2^ is very close to 1. Figure 7a shows the barrier height and ideality factor for the as-deposited sample and the sample with PAA as they were extracted at temperatures ranging from 300 K to 450 K. As the temperature increases, the barrier height increases and the ideality decreases, which is attributed to barrier inhomogeneities at the W/Au and Ga_2_O_3_ interface. The barrier inhomogeneities exhibited a strong temperature dependence process. At room temperature, carriers primarily cross regions with low barrier heights, often associated with defects at the W/Ga_2_O_3_ contact surface, resulting in a large ideality factor. However, as the temperature increased, the carriers gained sufficient energy to cross the higher barrier heights, leading to a smaller ideality factor. Furthermore, conventional Richardson’s plots *ln*(*I_s_*/*T*^2^) as a function of 1/*kT* were plotted for temperatures 300–450 K in Figure 7b. The barrier height *qФ_b_*_,0_ and the Richardson constant A** were extracted from the slope and intercept of the linear fit, respectively. For the as-deposited sample, the calculated *qФ_b_*_,0_ and A** are 0.69 eV and 0.082 A/cm^2^K^2^ respectively, while for the PAA-treated sample, these values are 0.76 eV and 0.481 A/cm^2^K^2^. The obtained Richardson constants are several orders of magnitude lower than the theoretical value of A** for *β*-Ga_2_O_3_. This discrepancy arises because the conventional Richardson plot assumes that the barrier height and ideality factor are temperature-independent, which is not valid in the presence of barrier inhomogeneities [25].

**Figure 8:**
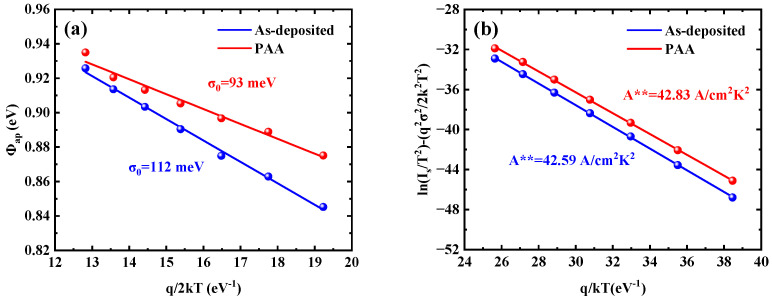
(**a**) The Φ_ap_ versus q/2kT plot and (**b**) the modified Richardson plot with the as-deposited sample and the sample with 450 °C PAA.

Thus, to explain the previously observed temperature dependence of barrier heights and ideality factors and the abnormal Richardson constants, barrier inhomogeneity was incorporated into the TE model in combination with a Gaussian distribution function for the barrier height, as expressed by the following Equation [39,40,41]:

(10) $$\Phi_{\mathrm{ap}}={\overset{-}{\Phi }}_{b0}-\frac{{q\sigma }_{0}^{2}}{2\mathrm{kT}}$$

where *Ф_ap_* is the apparent barrier height, *Ф_b_*_0_ and *σ*_0_ are the mean barrier height and standard deviation, respectively. Figure 8a shows the *Ф_ap_* versus *q*/2*kT* plot, and the *σ*_0_ was determined with the slope of the fit line. The obtained *σ*_0_ values are 112 meV for the as-deposited sample and 92 meV for the sample with PAA. This reduction in *σ*_0_ after post-anode annealing is attributed to the reduction of oxygen vacancies, which serve as low-barrier height patches on the W/Ga_2_O_3_ contact surface, thereby decreasing fluctuations in the barrier height. Furthermore, a modified Richardson plot was derived using the following Equation:

(11) $$\ln(\frac{I_{s}}{T^{2}})-\frac{q^{2}\sigma_{0}^{2}}{{2k}^{2}T^{2}}=\mathrm{lnA}A^{**}-\frac{q\Phi_{b0}}{\mathrm{kT}}$$

According to Equation (11), the modified Richardson plots are shown in Figure 8b. The modified Richardson constants were determined from the y-axis intercepts of the linear fits to the plots, yielding values of 42.59 A/cm^2^K^2^ for the as-deposited sample and 42.83 A/cm^2^K^2^ for the sample with PAA treatment. These values are close to the theoretical Richardson constant of 41.1 A/cm^2^K^2^. This result indicates that the barrier height of the W/Au-Ga_2_O_3_ Schottky contacts follows Gaussian distribution for both the as-deposited sample and the sample with PAA.

## 4. Conclusions

In conclusion, the electrical characteristics of *β*-Ga_2_O_3_ Schottky barrier diodes using W/Au as the Schottky metal were studied. Due to 450 °C post-anode annealing, the oxygen vacancy defects on the *β*-Ga_2_O_3_ surface were reduced, resulting in a decrease in the surface potential. The increase in barrier height and the reduction in leakage paths improved the characteristics of the W/Au-Ga_2_O_3_ SBDs, whereas the breakdown voltage increased by 56.25%, rising from 400 V to 625 V after PAA treatment. Additionally, the temperature dependence of barrier heights and ideality factors was observed over a temperature range of 300 K to 450 K, attributed to barrier inhomogeneity. The thermionic emission model combined with a Gaussian distribution of barrier heights was used to explain this temperature dependence. Post-annealing was found to reduce barrier height fluctuations from 112 meV to 92 meV. As evidenced by the modified Richardson constants of 42.59 A/cm^2^K^2^ for the as-deposited sample and 42.83 A/cm^2^K^2^ for the PAA-treated sample, both of which are very close to the theoretical Richardson constant for *β*-Ga_2_O_3_. These results demonstrate that the barrier inhomogeneity of W/Au-Ga_2_O_3_ SBDs can be effectively described using the TE model with a Gaussian distribution of barrier heights.

## Data Availability

The original contributions presented in the study are included in the article, further inquiries can be directed to the corresponding author.

## References

[1] Cassabois G., Valvin P., Gil B. (2016). Hexagonal boron nitride is an indirect bandgap semiconductor. Nat. Photonics.

[2] Hickman A.L., Chaudhuri R., Bader S.J., Nomoto K., Li L., Hwang J.C.M., Grace Xing H., Jena D. (2021). Next generation electronics on the ultrawide-bandgap aluminum nitride platform. Semicond. Sci. Technol..

[3] Xie L., Xu S., Zhang T., Tao H., Su H., Gao Y., Liu X., Zhang Y., Zhang J., Hao Y. (2025). Improved crystal quality of β-Ga_2_O_3_ on sapphire (0001) substrates by induced-nucleation technique and enhancement of Ga_2_O_3_ UV photodetectors performance. J. Alloys Compd..

[4] Sasama Y., Kageura T., Imura M., Watanabe K., Taniguchi T., Uchihashi T., Takahide Y. (2022). High-mobility p-channel wide-bandgap transistors based on hydrogen-terminated diamond/hexagonal boron nitride heterostructures. Nat. Electron..

[5] Butenko P.N., Boiko M.E., Guzilova L.I., Krymov V.M., Shapenkov S.V., Sharkov M.D., Verbitskii V.N., Zarichny A.A., Nikolaev V.I. (2024). Enhancing the perfection of bulk (100) β-Ga_2_O_3_ crystals grown by Czochralski method. J. Cryst. Growth.

[6] Sheoran H., Kumar V., Singh R. (2022). A Comprehensive Review on Recent Developments in Ohmic and Schottky Contacts on Ga_2_O_3_ for Device Applications. ACS Appl. Electron. Mater..

[7] Ahmed S.S., Islam A.E., Dryden D.M., Liddy K.J., Hendricks N.S., Moser N.A., Chabak K.D., Green A.J. (2024). Theoretical Power Figure-of-Merit in β-Ga_2_O_3_ Lateral Power Transistors Determined Using Physics-Based TCAD Simulation. IEEE Trans. Electron Devices.

[8] Zhang J., Li X., Zhu R., Wang K., Zhang B., Zhang C. (2024). Low loss and low EMI noise trench IGBT with shallow emitter trench controlled p-type dummy region. Chin. J. Electron..

[9] Liu A.-C., Hsieh C.-H., Langpoklakpam C., Singh K.J., Lee W.-C., Hsiao Y.-K., Horng R.-H., Kuo H.-C., Tu C.-C. (2022). State-of-the-Art β-Ga_2_O_3_ Field-Effect Transistors for Power Electronics. ACS Omega.

[10] Li X., Jiang W., Wang Y., Zhang H., Peng C., Zhang X., Liang X., Fu W., Zhang Z., Lei Z. (2024). Single-event burnout in β-Ga_2_O_3_ Schottky barrier diode induced by high-energy proton. Appl. Phys. Lett..

[11] Green A.J., Speck J., Xing G., Moens P., Allerstam F., Gumaelius K., Neyer T., Arias-Purdue A., Mehrotra V., Kuramata A. (2022). β-Gallium oxide power electronics. APL Mater..

[12] Green A.J., Chabak K.D., Baldini M., Moser N., Gilbert R., Fitch R.C., Wagner G., Galazka Z., Mccandless J., Crespo A. (2017). β-Ga_2_O_3_ MOSFETs for Radio Frequency Operation. IEEE Electron Device Lett..

[13] Wong M.H. (2023). A landscape of β-Ga_2_O_3_ Schottky power diodes. J. Semicond..

[14] Gammon P.M., Pérez-Tomás A., Shah V.A., Vavasour O., Donchev E., Pang J.S., Myronov M., Fisher C.A., Jennings M.R., Leadley D.R. (2013). Modelling the inhomogeneous SiC Schottky interface. J. Appl. Phys..

[15] Splith D., Müller S., Schmidt F., von Wenckstern H., van Rensburg J.J., Meyer W.E., Grundmann M. (2014). Determination of the mean and the homogeneous barrier height of Cu Schottky contacts on heteroepitaxial β-Ga_2_O_3_ thin films grown by pulsed laser deposition. Phys. Status Solidi A.

[16] Greco G., Giannazzo F., Fiorenza P., Di Franco S., Alberti A., Iucolano F., Cora I., Pecz B., Roccaforte F. (2018). Barrier Inhomogeneity of Ni Schottky Contacts to Bulk GaN. Phys. Status Solidi A.

[17] Lee K.Y., Huang Y.H. (2012). An Investigation on Barrier Inhomogeneities of 4H-SiC Schottky Barrier Diodes Induced by Surface Morphology and Traps. IEEE Trans. Electron Devices.

[18] Yadav M.K., Mondal A., Sharma S.K., Bag A. (2024). Unveiling Thermal Effects on Sn-Doped β-Ga_2_O_3_ Schottky Barrier Diodes on Sapphire for High-Temperature Power Electronics. IEEE Trans. Electron Devices.

[19] Hou C., Gazoni R.M., Reeves R.J., Allen M.W. (2019). Oxidized Metal Schottky Contacts on (010) β-Ga_2_O_3_. IEEE Electron Device Lett..

[20] Taşçıoğlu İ., Aydemir U., Altındal Ş. (2010). The explanation of barrier height inhomogeneities in Au/n-Si Schottky barrier diodes with organic thin interfacial layer. J. Appl. Phys..

[21] Triendl F., Pfusterschmied G., Pobegen G., Konrath J.P., Schmid U. (2020). Theoretical and experimental investigations of barrier height inhomogeneities in poly-Si/4H-SiC heterojunction diodes. Semicond. Sci. Technol..

[22] Reddy P.R.S., Janardhanam V., Shim K.-H., Reddy V.R., Lee S.-N., Park S.-J., Choi C.-J. (2020). Temperature-dependent Schottky barrier parameters of Ni/Au on n-type (001) β-Ga_2_O_3_ Schottky barrier diode. Vacuum.

[23] Higashiwaki M., Konishi K., Sasaki K., Goto K., Nomura K., Thieu Q.T., Togashi R., Murakami H., Kumagai Y., Monemar B. (2016). Temperature-dependent capacitance–voltage and current–voltage characteristics of Pt/Ga_2_O_3_ (001) Schottky barrier diodes fabricated on n^-^–Ga_2_O_3_ drift layers grown by halide vapor phase epitaxy. Appl. Phys. Lett..

[24] Hou C., Gazoni R.M., Reeves R.J., Allen M.W. (2021). Dramatic Improvement in the Rectifying Properties of Pd Schottky Contacts on β-Ga_2_O_3_ During Their High-Temperature Operation. IEEE Trans. Electron Devices.

[25] Iucolano F., Roccaforte F., Giannazzo F., Raineri V. (2007). Barrier inhomogeneity and electrical properties of Pt/GaN Schottky contacts. J. Appl. Phys..

[26] Garg M., Naik T.R., Pathak C.S., Nagarajan S., Rao V.R., Singh R. (2018). Significant improvement in the electrical characteristics of Schottky barrier diodes on molecularly modified Gallium Nitride surfaces. Appl. Phys. Lett..

[27] Qian L.-X., Wu Z.-H., Zhang Y.-Y., Lai P.T., Liu X.-Z., Li Y.-R. (2017). Ultrahigh-Responsivity, Rapid-Recovery, Solar-Blind Photodetector Based on Highly Nonstoichiometric Amorphous Gallium Oxide. ACS Photonics.

[28] Mi W., Ma J., Luan C., Xiao H. (2014). Structural and optical properties of *β*-Ga_2_O_3_ films deposited on MgAl_2_O_4_ (100) substrates by metal–organic chemical vapor deposition. J. Lumin..

[29] Polyakov A., Lee I.-H., Smirnov N., Shchemerov I., Vasilev A., Chernykh A., Pearton S. (2020). Electric field dependence of major electron trap emission in bulk *β*-Ga_2_O_3_: Poole–Frenkel effect versus phonon-assisted tunneling. J. Phys. D Appl. Phys..

[30] Yao Y., Davis R.F., Porter L.M. (2017). Investigation of Different Metals as Ohmic Contacts to *β*-Ga_2_O_3_: Comparison and Analysis of Electrical Behavior, Morphology, and Other Physical Properties. J. Electron. Mater..

[31] Zhang T., Lee C.-Y., Gong B., Lim S., Wenham S., Hoex B. (2018). In situ x-ray photoelectron emission analysis of the thermal stability of atomic layer deposited WO_x_ as hole-selective contacts for Si solar cells. J. Vac. Sci. Technol. A.

[32] Chen W.-J., Ma H.-P., Gu L., Shen Y., Yang R.-Y., Zhang J., Yang L., Zhu J., Zhang Q.-C. (2023). Influence of nitrogen annealing treatment on optical, microstructural, and chemical properties of Ga_2_O_3_ film grown by plasma-enhanced atomic layer deposition. J. Phys. Chem. C.

[33] Gao S., Yang X., Cheng J., Guo X., Kang R. (2023). Deformation and fracture behaviors of monocrystalline *β*-Ga_2_O_3_ characterized using indentation method and first-principles calculations. Mater. Charact..

[34] Hong Y., Zheng X., He Y., Liu K., Zhang H., Wang X., Yuan Z., Zhang F., Wang Y., Ma X. (2024). Enhancing performance of β-Ga_2_O_3_ diodes through a Ni_x_O/SiN_x_/Ga_2_O_3_ sandwich structure. J. Alloys Compd..

[35] Wang Z., Gong H.-H., Yu X.-X., Ji X., Ren F.-F., Yang Y., Gu S., Zheng Y., Zhang R., Ye J. (2023). Trap-mediated bipolar charge transport in NiO/Ga_2_O_3_ p^+^-n heterojunction power diodes. Sci. China Mater..

[36] Xian M., Fares C., Ren F., Gila B.P., Chen Y.-T., Liao Y.-T., Tadjer M., Pearton S.J. (2019). Effect of thermal annealing for W/β-Ga_2_O_3_ Schottky diodes up to 600 °C. J. Vac. Sci. Technol. B.

[37] Chen J., Bian Z., Liu Z., Zhu D., Duan X., Wu Y., Jia Y., Ning J., Zhang J., Hao Y. (2021). Effects of thermal annealing on the electrical and structural properties of Mo/Au schottky contacts on n-GaN. J. Alloys Compd..

[38] Tung R.T. (1992). Electron transport at metal-semiconductor interfaces: General theory. Phys. Rev. B.

[39] Subhash C., Jitendra K. (1997). Simulation and analysis of the I-V characteristics of a Schottky diode containing barrier inhomogeneities. Semicond. Sci. Technol..

[40] Chand S., Kumar J. (1997). Effects of barrier height distribution on the behavior of a Schottky diode. J. Appl. Phys..

[41] Pakma O., Serin N., Serin T., Altındal Ş. (2008). The double Gaussian distribution of barrier heights in Al/TiO_2_/p-Si (metal-insulator-semiconductor) structures at low temperatures. J. Appl. Phys..

